# Dental needs in palliative care and problems in dental hygienist education: survey study of palliative care ward homepage, university syllabus, and academic conference abstracts

**DOI:** 10.1186/s12904-022-01029-9

**Published:** 2022-07-30

**Authors:** Madoka Funahara, Sakiko Soutome, Mitsunobu Otsuru, Yuki Sakamoto, Hiromi Honda, Yumiko Ikegami, Nagato Natsume, Masahiro Umeda, Atsuko Nakamichi

**Affiliations:** 1 School of Oral Health Sciences, Kyushu Dental University, Fukuoka, Japan; 2 Department of Oral Health, Nagasaki University Graduate School of Biomedical Sciences, Nagasaki, Japan; 3 Department of Clinical Oral Oncology, Nagasaki University Graduate School of Biomedical Sciences, Nagasaki, Japan; 4 Department of Dentistry and Oral Surgery, Kansai Medical University Medical Center, Osaka, Japan; 5 Department of Nursing, Tokyo Metropolitan Cancer and Infectious Diseases Center Komagome Hospital, Tokyo, Japan; 6 Division of Research and Treatment for Oral and Maxillofacial Congenital Anomalies, School of Dentistry, Aichi Gakuin University, Aichi, Japan

**Keywords:** Palliative care, Dental hygienist, Education, University, Syllabus

## Abstract

**Background:**

Although end-of-life patients have a variety of oral-related symptoms, the involvement of dentists and dental hygienists in the palliative care teams is limited. This study investigates the current state of palliative care education in universities that train dentists and dental hygienists and the need for dentistry in the clinical setting of palliative medicine in Japan.

**Methods:**

First, we investigated the involvement of dentistry in hospitals with palliative care units from a website. The number of reports on palliative care presented by dental hygienists at academic conferences around 2016, when the public medical insurance system in Japan covered oral care for patients with terminal illnesses, were examined. We also surveyed the syllabuses of the university that trained nurses, dentists, and dental hygienists to determine their education regarding palliative care.

**Results:**

Of the 376 hospitals with palliative care units, 176 (46.8%) had dentistry in the hospital. Additionally, 321 hospitals (85.4%), which included those without dentistry, responded that they provided oral care by dentists and dental hygienists in the palliative care unit. There were only two presentations on palliative care in the annual meetings of the two major academic societies by dental hygienists between 2012 and 2016. However, this number increased rapidly to 47 between 2017 and 2020. The syllabus surveys showed that, compared to nursing universities, universities that trained dentists or dental hygienists had lesser education in palliative care. Furthermore, education in the universities that trained dental hygienists was mostly related to the oral care of patients with terminal illnesses, while the physical and mental conditions of end-of-life patients were not well educated.

**Conclusion:**

Considering that society requires the involvement of dental hygienists in the field of palliative care, it is necessary to enhance basic and clinical education of palliative care in universities that train dentists and dental hygienists to provide good oral care to patients with terminal illnesses and contribute to improving their quality of life.

## Background

The World Health Organization (WHO) defined palliative care in 2002 as an approach that improves the quality of life of patients and their families facing the problems associated with a life-threatening illness and described that this was achieved through the prevention and relief of suffering by means of early identification and impeccable assessment and treatment [1]. However, the timing of referrals to palliative care units tends to be late or very late in Japan from the families’ perspectives according to the survey of Morita et al. [2]. In Japan, the Cancer Control Act, approved in 2006, aimed to promote cancer control comprehensively and systematically. Based on this act, the Basic Plan to Promote Cancer Control program was approved by the Ministry of Health, Labour and Welfare in 2007 [3]; accordingly, the national and local governments would ensure that palliative care is provided appropriately. The care included relief of physical symptoms, such as pain associated with cancer, and assistance for psychological problems, not only at the end-of-life but also during the initial stage of the treatment. Additionally, the enhancement of home medical care such that patients could live their lives while receiving medical treatment both in the hospital and at home was included. Furthermore, the plan promoted educational institutions, such as universities, to formulate practical educational programs for palliative care that incorporated practical training, and the establishment of a palliative medicine course in medical schools to train educational leaders who would be responsible for the pre-graduation education of doctors.

In Japan’s medical and nursing education, the model core curriculum, which shows the scope of questions for national examinations, includes the item “palliative care” as an essential item. Consequently, in 2015, 98.5% of the Japanese medical schools gave lectures on palliative care as reported by Nakamura et al. [4]. Nagasawa et al. compared the results of palliative care knowledge tests conducted in 2008 and 2015 for doctors throughout Japan and reported that the palliative care education program was successful [5].

There are many oral problems such as dry mouth, mouth ulcers, oral candidiasis, feeding difficulties, and dysphagia during cancer treatment and care [6]. Therefore, dentists and dental hygienists are required to act [7]. However, universities that train Japanese dentists or dental hygienists do not include items related to palliative care in the scope of the questions in the national examination. To date, no studies have investigated the extent to which dentists and dental hygienists are educated in palliative care at universities. In 2012, the public health insurance system in Japan allowed dentists and dental hygienists to provide professional oral care for terminal patients. However, no investigation has been conducted on how much dentists and dental hygienists are actually involved in the field of palliative care. Therefore, this study investigated the actual situation of palliative care education in universities that trained dentists and dental hygienists and the need for dentistry in the clinical setting of palliative medicine.

## Methods

### Design and setting

This study conducted a survey of hospital homepages, university syllabuses, and academic journals in Japan to investigate the involvement of dentistry in palliative care education in Japan. Furthermore, a questionnaire survey on palliative care was conducted for students of one dental hygienist training university. Palliative care in this paper refers to the stage of the disease where active treatment for cancer is completed and only palliative medicine is performed.

### Survey of providing oral care by dentists and dental hygienists in hospitals with palliative care units (performed by SS, MO, and YS)

The status of dental care provision in hospitals with palliative care units was investigated from the website of Hospice Palliative Care Japan (https://hpcj.org, accessed on August 27, 2021), the largest association in palliative care in Japan. We investigated whether each hospital had dentistry and whether it was possible to receive dentistry services through home visits.

### Survey of the conference presentation by dental hygienists (performed by SS MO, YS, and HH)

We investigated the number of presentations on palliative care by dental hygienists between 2012 and 2020 in the nationwide annual academic conferences of the Japanese Society for Palliative Medicine or the Japanese Society of Oral Care from the society’s program (surveyed on August 27, 2021).

### Survey of the syllabus in universities that trained nurses, dentists, and dental hygienists (performed by MF, and HH)

The syllabuses in 2017 and 2021 of universities that trained nurses, dentists, and dental hygienists were examined from the homepage of each university to determine whether there was an education in palliative care (accessed on August 15, 2021). We searched for the words “palliative medicine”, “palliative care”, “death and life”, “terminal stage”, “terminal”, “end-of-life”, and “spiritual care”. Courses that had these words were defined as courses related to palliative care, and lectures that were not included in the course but contained these words in another course, such as oral surgery or geriatric dentistry, were defined as lectures related to palliative care. Palliative care and hospital training were also investigated. Furthermore, the contents of the syllabus of the dental hygienist training university were examined in detail.

### Questionnaire survey on palliative care for the student in a university that trained dental hygienists (performed by MF, and HH)

We conducted a questionnaire survey on students’ attitudes toward palliative care. The questionnaire was conducted as a preliminary survey for students of the university to which the first author belongs. Therefore, no sample size calculation or pre-check of the questionnaire was not performed. The subjects were second to fourth grade students from a university (University E in Table 3), where education in palliative care was relatively substantial. The questionnaire is exploratory using a small number of cases. The questionnaire task was that the involvement of dental hygienists was small in the clinical practice of palliative care. The hypothesis was that palliative care education was insufficient in the education of dental hygienists. The method was to hand over the questionnaire form anonymously and collect it before the lecture. The answers to the following eight questions were tabulated: 1) Are you motivated to provide palliative care? 2) Did you have a lecture on palliative care at the university? 3) Can the knowledge gained in the lecture be applied clinically? 4) Are university lectures on palliative care enough? 5) What would you want to add to the university palliative care education? (multiple answers allowed); lecture/exercise/hospital training 6) Do you think the social needs of dental hygienists in palliative care will increase? 7) Do you think you will be involved in end-of-life patients in the future? and 8) The terms you know (multiple answers allowed); mental spiciness/oral care for end-of-life patients/angel care/grief care/spiritual pain/death care/thanatology.

## Results

There were 453 hospitals with palliative care units in Japan (as of November 2020), of which 376 (83%) were able to collect information from the website of Hospice Palliative Care Japan (https://hpcj.org) (Table 1). Of these, 176 (46.8%) had dentistry and 321 hospitals (85.4%), which included those without dentistry, responded that they could provide oral care from dentists and dental hygienists in the palliative care unit. In hospitals that do not have dentistry, dentists and dental hygienists visit the palliative care unit to provide oral care.

**Table 1:** Dentistry in the hospital and availability of oral care by a dentist or dental hygienist

		Number of hospitals
Dentistry in the hospital	Yes	176 (46.8%)
	No	200 (53.2%)
Oral care by dentist or dental hygienist in the palliative care unit	Available	321 (85.4%)
	Unavailable	55 (14.6%)
Total		376

Oral care by dentists and dental hygienists for patients with terminal illnesses was covered by public medical insurance since 2016. There were two presentations on palliative care in the annual meetings of the two academic societies by dental hygienists between 2012 and 2016, while the number increased rapidly to 47 between 2017 and 2020. All of these were clinical research presentations, with no basic research or educational presentations.

A survey of the syllabus of university training nurses, dentists, and dental hygienists is shown in Table 2. A total of 11 national universities in Japan had faculty members who trained nurses. Of these, eight universities browsed the syllabus in 2017. There were six courses in palliative care at five universities, 20 lectures on palliative care at six universities, 18 h of palliative care exercises at two universities, and hospital training at five universities. In 2021, 11 universities were able to view the syllabus; there were six courses and 56 lectures on palliative care, 26 h of palliative care exercises at four universities, and hospital training at seven universities.

**Table 2:** Education in palliative care in universities training nurses, dentists, and dental hygienists

	2017	2021
Nursing course	8 universities	11 universities
Number of courses on palliative care	6 courses / 5 universities	6 courses / 6 universities
Number of lectures on palliative care	20 lectures / 6 universities	56 lectures / 11 universities
Hours of palliative care exercise(s)	18 h / 2 universities	26 houres / 4 universities
Hospital training	5 universities	11 universities
Dentist course	10 universities	12 universities
Number of courses on palliative care	1 course / 1 university	1 course / 1 university
Number of lectures on palliative care	12 lectures / 6 universities	20 lectures / 7 universities
Hours of palliative care exercise(s)	0 h	0 h
Hospital training	1 universities	2 universities
Dental hygienist course	6 universities	7 universities
Number of courses on palliative care	0 courses	0 courses
Number of lectures on palliative care	11 lectures / 5 universities	12 lectures / 5 universities
Hours of palliative care exercise(s)	1 h / 1 university	1 h / 1 university
Hospital training	1 university	1 university

There were 12 national and public universities in Japan that had a faculty of dentistry. Of these, 10 universities browsed the syllabus in 2017. There was one course on palliative care at one university and 12 lectures at six universities. None of the universities conducted palliative care exercises, and only one university organized hospital training. Of the 12 universities that viewed the 2021 syllabus, there was one course for palliative care, and 20 lectures were given at seven universities. None of the universities conducted palliative care exercises, and two universities offered hospital training.

A total of seven national and public universities in Japan had a faculty of dental hygienists, of which six browsed the syllabus in 2017. None of the universities had a course on palliative care, and there were 11 lectures in five universities. A university conducted palliative care exercises, and another offered hospital training. There were no courses on palliative care at the seven universities that viewed the syllabus in 2021, while there were 11 lectures at five universities. A university conducted palliative care exercises and another offered hospital training.

These syllabus surveys showed that compared to nursing universities, those that trained dentists or dental hygienists had lesser education in palliative care, and there was not much difference between 2017 and 2021.

We investigated the syllabus of the five dental hygienist training universities that gave lectures on palliative medicine in 2017 and 2021 (Table 3) in further detail. A total of nine items, which comprised eight items on palliative care in the model core curriculum of nursing education, and one on the oral symptoms of patients who were terminally ill, were examined to determine whether they were taught in the universities of dental hygienists’ training. In 2021, three out of five schools gave lectures on oral adverse events that appeared during cancer treatment or at end-of-life, and oral care methods. In addition, lectures on views of life and death and contents related to pain evaluation were offered in two schools. However, physical changes in end-of-life stage patients, which were essential for oral care, were not included in the lecture content, and education in collaboration with related organizations and other occupations, and on decision-making processes, grief care, post-mortem care, etc., were also shown to be inadequate.

**Table 3:** Educational content on palliative care in universities training dental hygienists

Item	University A		University B		University C		University D		University E	
	2017	2019	2017	2019	2017	2019	2017	2019	2017	2019
Oral adverse events	〇	〇	〇	〇	ー	ー	ー	ー	〇	〇
Physical changes in people in the final stages of life	ー	ー	ー	ー	ー	ー	ー	ー	ー	ー
Thanatology	ー	〇	ー	ー	ー	ー	〇	ー	〇	〇
The importance of liaising with relevant agencies and professions	ー	ー	ー	ー	ー	ー	ー	ー	ー	ー
Methods of assessment, control of pain, and total care for pain relief	ー	ー	ー	ー	〇	〇	ー	ー	ー	〇
Acceptance process of death and spiritual care of the family	ー	ー	ー	ー	ー	ー	ー	〇	〇	ー
The decision-making process	ー	ー	ー	ー	ー	ー	〇	ー	ー	ー
Family care after death and grief care	ー	ー	ー	ー	ー	ー	ー	ー	ー	〇
The significance of care after death	ー	ー	ー	ー	ー	ー	ー	ー	ー	ー

Table 4 shows the results of the questionnaire administered to students from a university training dental hygienist. Most students were motivated for palliative care, and more so in the third and fourth grades compared to the second grade, although statistical examination could not be done since the number of cases was small in the preliminary survey of only one school. Students thought that the knowledge gained in the lectures on palliative care could be applied to clinical practice; however, the majority of the third and fourth grades felt that palliative care education was inadequate. Most students believed that society required the involvement of dental hygienists in palliative care. However, it was also revealed that they lacked knowledge regarding angel care, grief care, spiritual pain, death care, and thanatology.

**Table 4:** Questionnaire survey on palliative care for students from a university training dental hygienists

Question	Number (excluding unknown)		
	2nd grade	3rd grade	4th grade
1. Are you motivated for palliative care?			
Yes	19	45	22
No	6	4	2
2. Did you take a lecture on palliative care at the university?			
Yes	21	29	23
No	4	20	1
3. Can the knowledge gained in the lecture be applied clinically?			
Yes	20	27	20
No	1	2	3
4. Are university lectures on palliative care enough?			
Yes	11	21	10
No	4	24	14
5. What would you want to add to the university palliative care education? (multiple answers allowed)			
Lecture	13	27	15
Exercise	12	21	11
Hospital training	17	33	19
6. Do you think the social needs of dental hygienists in palliative care will increase?			
Yes	21	47	24
No	4	2	0
7. Do you think you will be involved in end-of-life patients in the future?			
Yes	16	40	13
No	9	9	11
8. The terms you know (multiple answers allowed)			
Mental spiciness	15	37	20
Oral care for end-of-life patients	14	35	23
Angel care	1	29	9
Grief care	0	11	5
Spiritual pain	2	15	4
Death care	8	42	0
Thanatology	2	2	0
Total number of participants	25	49	24

## Discussion

This study showed that pre-graduate education related to palliative care was inadequate in universities that trained dentists or dental hygienists compared to universities that trained nurses.

Many oral problems are reported in patients who are terminally ill. Therefore, dentists and dental hygienists are required to participate in palliative care teams in clinical practice. Wilberg et al. reported that the frequency of dry mouth, mouth pain, problems with food intake, oral candidiasis, and rich dental plaque were 78%, 67%, 56%, 34%, and 24%, respectively, in 99 patients with terminal cancer [8]. In a systematic review, Venkatasalu et al. found that xerostomia, oral candidiasis, and dysphagia were the three most common oral conditions among palliative patients, followed by mucositis, orofacial pain, taste change, and ulceration [6]. Professional oral care is a very important supportive care to provide quality care at end of life, and we think that it is important to clarify the current status of the integration of palliative medicine and dentistry.

In 1990, Brown et al. emphasized the importance of dental hygienists in an interdisciplinary team for palliative care, whose roles were educators, dental consultants, and primary care providers [9]. Ohno et al. stated that considering the high incidence of oral complications in patients with terminal cancer, dental services were necessary for high-quality palliative care, but there was insufficient availability [10]. Mol also reported that a trained dentist would be a good teammate for an oncologist, radiation therapist, or other doctors of a palliative care team [11]. According to this study, 47% of the hospitals with palliative care wards had dentistry, and 85.4%, which included hospitals without dentistry, could provide oral care by dentists and dental hygienists in the ward. Furthermore, since 2016, when oral care by dentists and dental hygienists for patients with a terminal illness has been covered by public medical insurance in Japan, the number of presentations on palliative care by dental hygienists at academic conferences has increased.

As described above, the participation of dentists and dental hygienists is required in the medical field of palliative care, and in fact, some dentists and dental hygienists should work as members of the palliative care team. Nevertheless, this study showed that both the extent and quality of palliative care education in universities that trained dentists and dental hygienists were currently extremely low. The nursing education model core curriculum (2017 revised edition) requires students to learn the following eight items related to palliative care: 1) Physical changes in people in the final stages of life, 2) Values, the outlook of life and death (Thanatology), 3) The importance of liaising with relevant agencies and professions, 4) Methods of assessment, control of pain, and total care for pain relief, 5) Acceptance process of death and spiritual care of the family, 6) The decision-making process, 7) Family care after death and grief care, and 8) The significance of care after death. However, this survey revealed that few dental hygienist training universities provide education on these contents [12]. Although lectures and practices on oral care in patients with terminal illness were given at some universities training dental hygienists, we believe that oral care of good quality cannot be provided without understanding the physical and mental conditions of end-of-life patients. These differences in the education of nurses and dental hygienists are thought to be because the scope of questions in the national examination includes palliative care in the former but not in the latter.

Education is conducted according to the model core curriculum at Japanese universities. The model core curriculum is defined by the Ministry of Education, Culture, Sports, Science, and Technology, and is an extraction of the core part that should be tackled in common by all universities. In addition, questions will be asked from this model core curriculum during national examinations for doctors, dentists, nurses, etc. Currently, the model core curriculum of medical education and nursing education includes a description of palliative medicine, but that of dentistry education does not contain a description of palliative medicine. Furthermore, the model core curriculum itself has not yet been formulated for dental hygienist education. In Japan, dental hygienists became a national qualification in 1948. The education period was required to be at least 2 years in 1983, and at least 3 years in 2010. Currently, there are 12 four-year universities, 16 junior colleges, and 146 vocational schools. Most dental hygienists are still educated in vocational schools. To enhance hygienist education, we believe that it is necessary to first enhance palliative care education at a four-year university and formulate a model core curriculum for that purpose.

This study showed that students from a university training dental hygienists were highly interested in palliative care, but felt that current palliative care education was inadequate, especially in the upper grades. It was also found that most of the education at universities training dental hygienists was related to the oral care of terminal patients and that the physical and mental conditions of end-of-life patients were not well educated. Many students were unaware of terms such as Angel care, grief care, spiritual pain, death care, and thanatology. To provide good oral care to patients who are terminally ill and improve their quality of life, this study suggests that it is necessary to enhance basic and clinical education on palliative care in universities that train dentists and dental hygienists.

This study has some limitations. First, we only examined the syllabus of universities and did not investigate its contents, so it is unclear whether the results obtained can be generalized. Second, dental hygienist training schools comprise not only universities but also junior colleges and vocational schools, but this study did not cover all dental hygienist education. However, since palliative care education is inadequate even in 4-year universities, which are considered to have the most extensive educational content, it can be inferred that palliative care education is also not sufficient in junior colleges and vocational schools. There is no selection by researchers because we examined all that were confirmed on the website, but selection bias may have occurred because it did not include those that were not posted on the website. In addition, since the questionnaire is a preliminary study only for students of the university to which the first author belongs, it may not reflect the intentions of all students. It is the first to investigate the current state of palliative care education in Japanese universities training dentists and dental hygienists. The participation of dentists and dental hygienists in a palliative care team is an urgent issue, and we think it is necessary to provide sufficient education in palliative medicine and care at universities training dentists and dental hygienists.

## Conclusion

This study showed that both the extent and quality of palliative care education at universities that trained dentists and dental hygienists were currently extremely low. Considering that society requires the involvement of dental hygienists in the field of palliative care, it is necessary to enhance basic and clinical education on palliative care at universities training dentists and dental hygienists to provide good oral care to patients who are terminally ill and improve their quality of life.

## Data Availability

The datasets used and/or analyzed during the current study are available from the corresponding author upon reasonable request.
